# Whole-Genome Sequences of Listeria monocytogenes Sequence Type 6 Isolates Associated with a Large Foodborne Outbreak in South Africa, 2017 to 2018

**DOI:** 10.1128/genomeA.00538-18

**Published:** 2018-06-21

**Authors:** Mushal Allam, Nomsa Tau, Shannon L. Smouse, Phillip S. Mtshali, Florah Mnyameni, Zamantungwa T. H. Khumalo, Arshad Ismail, Nevashan Govender, Juno Thomas, Anthony M. Smith

**Affiliations:** a National Institute for Communicable Diseases, National Health Laboratory Service, Johannesburg, South Africa

## Abstract

We report whole-genome sequences for 10 Listeria monocytogenes sequence type 6 isolates associated with a large listeriosis outbreak in South Africa, which occurred over the period of 2017 to 2018. The possibility of listeriosis spreading beyond South Africa’s borders as a result of exported contaminated food products prompted us to make the genome sequences publicly available.

## GENOME ANNOUNCEMENT

Listeria monocytogenes is a Gram-positive bacterium that causes listeriosis. Acquisition of the pathogen occurs mainly by consumption of contaminated food. Infections with L. monocytogenes can result in mild febrile gastroenteritis in healthy individuals; however, invasive diseases such as bacteremia, meningitis, pneumonia, endocarditis, and sepsis can occur in high-risk groups (1).

In South Africa, a large, multiprovince outbreak of listeriosis caused by L. monocytogenes began in 2017. As of 27 April 2018, a total of 1,019 laboratory-confirmed clinical listeriosis cases were reported to the National Institute for Communicable Diseases, South Africa (http://www.nicd.ac.za/index.php/nicd-listeriosis-situation-report-27-april-2018). Multilocus sequence typing (MLST) of 521 clinical isolates using a whole-genome sequencing approach determined that 443 of 521 (85%) isolates belonged to L. monocytogenes sequence type 6 (ST6). Isolates of the same sequence type were found in a widely consumed ready-to-eat processed meat product called “polony” and in the processing environment (production facility) of the manufacturer of this implicated meat product. Here, we provide 10 whole-genome sequences of the ST6 outbreak strain representing isolates from three different source types (human, environmental, and food).

Standard microbiological techniques were performed to confirm identification of L. monocytogenes. Genomic DNA from each isolate was extracted using the QIAamp DNA minikit (Qiagen, Germany), and paired-end libraries were prepared using the Nextera XT DNA library kit, followed by 2 × 300-bp sequencing on a MiSeq platform (Illumina, Inc., USA). The paired-end reads were quality trimmed using Sickle version 1.33 and *de novo* assembled using SPAdes version 3.11 (2).

For the 10 isolates, genome assemblies contained an average of 161 contig sequences longer than 200 bp and covered an average total of 3,037,729 bp. The contigs for each isolate were then submitted to GenBank, where gene annotations were implemented using the NCBI Prokaryotic Genome Annotation Pipeline (PGAP) (3). The average total number of 3,207 genes predicted by PGAP includes 3,085 protein-coding genes, 36 pseudogenes, and 86 RNA genes on average. Key genomic features for the 10 isolates are summarized in Table 1.

**TABLE 1:** Isolate information and key genomic features of 10 L. monocytogenes whole-genome sequences from the 2017 to 2018 outbreak in South Africa

Isolate reference no.	Source	GenBank accession no.	No. of contigs	Size (bp)	No. of coding genes	No. of RNAs	No. of pseudogenes	Total no. of genes
HM00110618	Production facility	QEXB00000000	333	3,098,720	3,255	78	44	3,377
YA00078529	Human blood culture	QEXC00000000	101	3,081,235	3,112	83	43	3,238
YA00071388	Human blood culture	QEXD00000000	222	2,972,956	3,034	60	31	3,125
YA00082404	Food	QEXE00000000	116	3,078,799	3,111	77	33	3,221
YA00079283	Human throat swab	QEXF00000000	83	3,085,415	3,107	80	41	3,228
YA00072733	Human blood culture	QEXG00000000	255	2,974,005	3,052	57	28	3,137
YA00066964	Human blood culture	QEXH00000000	124	3,028,045	3,044	114	36	3,194
YA00075880	Human blood culture	QEXI00000000	166	3,016,857	3,044	121	35	3,200
IG01149260	Human blood culture	QEXJ00000000	98	3,023,744	3,048	95	36	3,179
HM00108598	Food	QEXK00000000	114	3,017,512	3,039	99	35	3,173

### Accession number(s).

The draft genome sequences reported here have been deposited at NCBI/GenBank under the accession numbers QEXB00000000 to QEXK00000000 (BioProject number PRJNA451422, BioSample numbers SAMN08970424 to SAMN08970415, respectively).
